# Map Changes and Theme Evolution in Work Hours: A Co-Word Analysis

**DOI:** 10.3390/ijerph15051039

**Published:** 2018-05-22

**Authors:** Bei Liu, Hong Chen, Xinru Huang

**Affiliations:** Department of Management School, China University of Mining and Technology, 1 Daxue Rd., Xuzhou 221116, China; liubeii@163.com (B.L.); hxr881003@163.com (X.H.)

**Keywords:** work hours, co-word analysis, theme evolution, individual–organization–society integrative perspective

## Abstract

(1) Background: Work hours are the basic carrier impacting employees’ work–life experience and organizational performance, and employees have greater anxiety in relation to work hours as new technology requires an increasingly faster work rhythm. However, scientific research on this topic lags far behind the practice, calling to attention the need for research on work hours from the perspective of historical evolution; (2) Methods: The Bibliometric method is used to analyze the 6364 articles and their contained 77 high-frequency keywords related to work hours from the Web of Science published between 1901 and 2017. Additionally, an individual–organization–society integrative perspective was adopted to describe the map changes and theme evolution of work hours; (3) Results and conclusions: The hot spots of research at the organizational level changed significantly around 1990, with the theme of “long work hours” becoming the core issue in recent years. Studies on the individual level have gradually moved from physiological aspects to the issues of burnout and psychological distress. Research topics related to the social level are somewhat loose, and mainly focused on work–life conflict areas. In addition, the cluster analysis based on the high-frequency keywords classifies six research types according to their research themes. Based on these findings, future trends are proposed to provide theoretical and practical reference for future studies.

## 1. Introduction

Work hours are a time-honored concept serving as an important instrument for valuing commodities because of work hours’ identity and measurability. Indexes related to time, such as “being late”, “early retreat” and “absence”, are also used to judge individual performance [1]. Since a reasonable work schedule could powerfully ensure particular organizational performance [2,3], the controversial concept of work hours between organizations and individuals has been proposed. To be specific, the simplest and straightest manner of pursuing profits is extending the employees’ work hours unboundedly, so as to obtain more surplus value. This notion is found in Adam Smith’s early theory of “Economic Man”, accompanied with extremely mechanistic and biologically philosophical colors, in regarding individuals as the robots. Furthermore, the “theory X” proposed by Douglas McGregor also suggests that organizations should simply push or force individuals to work for organizational goals because individuals’ only motivation is to obtain economic rewards [4]. Unsurprisingly, those notions facilitated the phenomenon that individuals extended work hours to cater to organizational requirements, either voluntarily or involuntarily.

With humans entering the Industry 4.0 [5], the traditional manufacturing industry has begun to realize intelligent transformation by tapping into the Internet of Things and big data, both of which present new dynamics to staff members’ physical and temporal requirements. Meanwhile, individuals’ work concept has also changed significantly, especially millennial-generation employees who tend to value time freedom more than prior generations [6,7]. However, the paradox is that compared with factors that may shake the absolute position of work hours, the organizational requirement for extending work hours has become more common, and meanwhile, the negative influence of long work hours has also emerged. Some previous research has demonstrated that the long work hours would stimulate more severe occupational injuries [8], affect employees’ work efficiency [9], decrease individuals’ physical and psychological health [10,11], and even cause family conflict [12]. In addition, long work hours have also become a critical factor in employees’ risk of suicide [13]. Evidence suggests that long work hours are detrimental to individual development and even encroach on the long-term interests of the organization (by disturbing the rhythm of individuals’ lives). These real impacts not only establish higher requirements for management practice, but also make it more urgent to grasp the historical context of work hours. Therefore, it is necessary to answer: Which changes have appeared on the maps of work hours given social and economic development? What were the main research themes at different stages? And, given these factors, what will the evolutionary tendency be? Descriptive analysis of the previous research on work hours can help answer these questions.

Most traditional research on work hours has been smaller and cross-sectional, exploring the influence of work hours on individuals or organizations. The classic study paradigm is the day reconstruction method. With this method, researchers conduct series analysis using subjects’ recorded activities on a particular day(s) or other fixed time periods [14,15]. Few researchers have discussed the trends of work hours. For example, Rones, Ilg and Gardner investigated the variation tendency of the average work hours in the United States during 1976–1993 [16]; Kuhn and Lozano explored the relationship between individual incomes and work hours in 1979–2006 based on data from the American Community Survey [17]; and there have also been studies in which different work hours were compared from a national perspective. For example, Aliaj, Flawinne, Jousten, Perelman and Shi compared work-hour variation tendencies for employees over 50 years old in Belgium, France, Germany and The Netherlands, from 1997 to 2011 [18]. However, these studies focused only on the specific number of work-hours changes, and failed to describe the research theme change on the map of work hours from an evolutionary aspect, let alone depict the evolutionary process of work hours. By directly assessing these aspects of work hours, this paper presents the development posture and the future course of the research on work hours based on prior studies. Thus, we may sketch the systematic context of the research on work hours to connect classic and emerging research themes, and expand the theoretical cognition of this field on the one hand, and on the other hand, provide an important reference for organizations to schematize a rational work schedule to promote the staff members’ physical and mental health.

## 2. Materials and Methods

### 2.1. Methodology

The co-word analysis method is a form of content analysis consisting of a co-word matrix which uses a statistic on occurrence frequency between particular keywords in the same object (e.g., literature, patents, web page, etc.) to reveal the affinity between the keywords and to analyze the subject and theme-structure changes [19]. This method’s basic assumptions are as follows: A keyword or a subject would represent the research theme if it appeared repeatedly in the related literature because it refined and concentrated the core content of the article [20]. Therefore, we may regard two articles as having a similar research subject concept, theory or method if the articles contained the same keywords; in addition, the more similar the keywords are, the closer the distance. Hoz-Correa adopted it to analyze the evolution of medical tourism [21]; Guo and her colleagues also used this method to analyze the research structure and future trends of organizational constraints [22].

Cluster analysis is a multivariate statistical analysis method for quantitative classification of multiple samples [23]. The basic analytic approach of this method is structuring a similar matrix based on statistics which could indicate the distance among samples or indexes from the multiple observations, and then dividing the observations into several clusters based on the measured distances, to grasp the structure and profile of observations. Therefore, we introduced the cluster analysis into the process of combing and summarizing the work-hours research to further explore the main categories of work hours and their relationship(s).

### 2.2. Data Procedure

To choose the main keywords related to work hours, we selected the largest international literature database—Web of Science—with querying “Science Citation Index Expanded” and “Social Science Citation Index”, and we set “article” and “review” as the document types. In 1901, a proposal named “An Act to Make Eight Hours a Legal Day’s Work for Mechanics, Workmen and Laborers” was presented to the United States Commonwealth of Massachusetts; this piece was regarded as early evidence of a focus on work hours, and thus we selected 1901–2017 as the study period. What is more, we did not set a specific category for this retrieval to improve the recall ratio. The keywords “work hours”, “work hour”, “the time of work”, “worked hour”, “work time” and “working hours” were used in the literature research on work hours, and in accordance with the Web of Science’s phrase-matching rules, we selected the classical forms “work time” and “work hour” as the keywords to obtain the most extensive information.

This paper utilized Bibexcel, an international scientometrics instrument developed in Sweden by Persson [24]. Bibexcel’s main features include bibliometrics, citation analysis, co-citation and cluster analysis. Additionally, we cleaned our results before analyzing the high-frequency keywords: First, we deleted the irrelevant keywords such as “work memory”; second, we combined and renamed some keywords due to them having similar meanings. For instance, the keywords “ hours of work”, “work hour”, “the time of work”, “worked hour”, “work time” and “working hours” were renamed “work hours”; moreover, “residency surgical”, “resident”, “junior residency” and “doctor”, and other words related to doctors, were renamed “doctors”, and the last 6364 articles were obtained finally.

## 3. Results

### 3.1. Publication Status of Work-Hours Research

**Figure 1:**
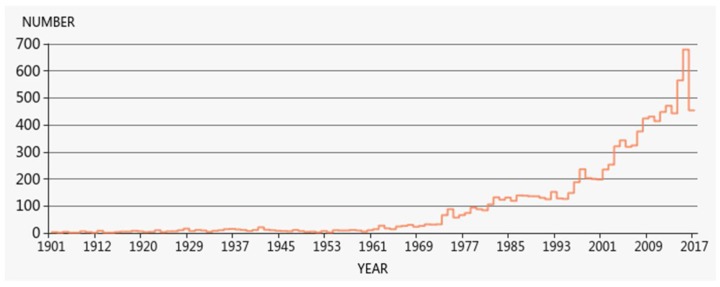
Annual number of selected articles related to work hours.

Figure 1 provides descriptive statistics for the number of research papers on work hours over the years. The number of studies shows an upward trend, with especially large growth after 2000. Of note, for 2017, we selected documents which were published/accessible before October 2017; thus, our scope does not include all potential documents in 2017.

Table 1 only shows the journals publishing more than 50 articles related to work hours, due to the large and imbalanced range of journals (and most periodicals were concentrated in medicine, sociology and management). In addition, the British Medical Journal (*n* = 210) ranked the first of this field issue, and the relevant research journals followed by the British Medical Journal were focused on labor issues, named as International Labour Review (*n* = 156) and Monthly Labor Review (*n* = 97).

### 3.2. The Co-Occurrence Analysis of High-Frequency Keywords

We selected 5781 keywords from the 6364 documents after deleting the irrelevant keywords and integrating the synonyms as the basis for the co-word analysis. We also adopted the following model to choose high-frequency keywords [25]: In the statistical results, the number “1” is 3001, and we had 77 keywords after the calculation. Table 2 lists the top 20 high-frequency vocabularies.

(1) $$N=\frac{1}{2}\left(-1\pm \sqrt{1+8I_{1}}\right)$$

Note. N represents the number of high-frequency keywords, $I_{1}$ represents the number of keywords that occurred only once.

**Figure 2:**
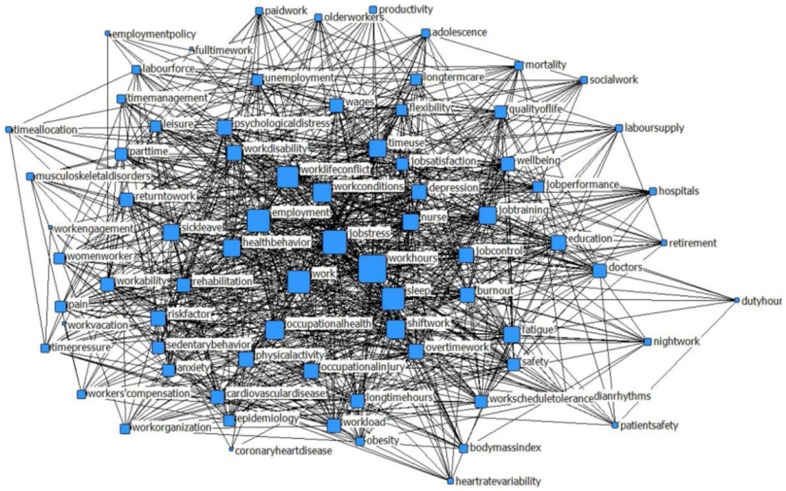
Co-occurrence network of high-frequency keywords.

NetDraw, with its visualized graphical function, developed by Professor Steve Borgatti, is a commonly used social network analysis program. According to the Bibexcel results, we constructed the co-occurrence matrix with 85 × 85 of work hours, and drew them into Netdraw to get the social network diagram of the high-frequency keyword (Figure 2).

In Figure 2, the size of blocks is directly proportional to their cited frequency—the larger the color block, the higher the frequency, with the lines connecting those keywords demonstrating the co-occurrence relationship between keywords. Keywords such as work, job condition, job stress and work–life conflict are the core themes in work-hours research (Figure 2).

The research results on the hypothesis of human nature, such as “economic man”, “social man” and “self-actualization man” put the relationship among the individual, organization and society into the focus of management [26], also providing new inspiration for the hierarchical analysis of management problems. The present study analyzed the historical literature in work hours based on the perspective of individual–organization–society integration, and we classified the present contents and outlined them based on time clues.

Note. The words “*bold and italic*” are new research issue spots emerging in different periods of time. The numbers in brackets indicate the number of published articles

**Figure 3:**
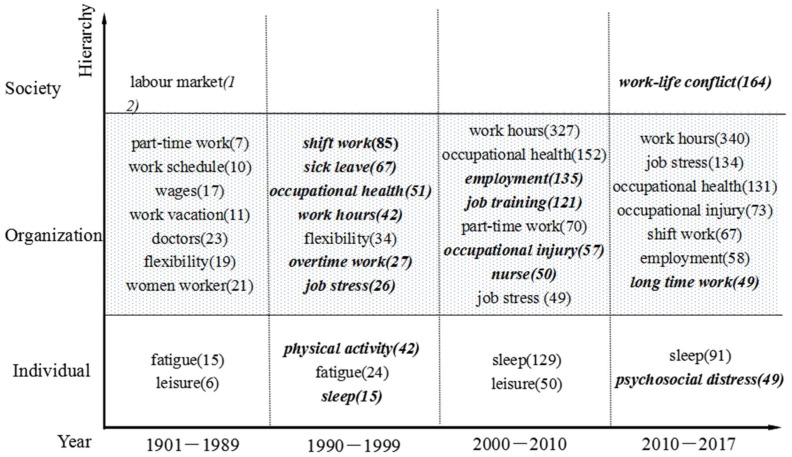
The specific time period of the keyword hierarchy.

Figure 3 shows the themes changes in the map of work hours. Due to the few studies on work hours existing before the 1890s, we unified these into the “1901–1989” period. The results illustrate that, from the perspective of individual–organization–society integration, the related research mainly focuses on the organizational level, followed by the individual level, while social studies are sparse. From the timeline perspective, the themes map of work hours changed significantly around 1990; this map has changed from the traditional economy to individual–society field, specifically, it has focused on employees’ work experience such as occupation health, job stress and overtime work, rather than wage, vacation and work-centered, which have not been explored until recently. Work conditions, job stress, occupational injury and nurse emerged after 2000, while the long work hours and work–life conflict were not transformed into new research themes until 2010.

### 3.3. Cluster Analysis of High-Frequency Keywords

According to the co-occurrence matrix, the higher frequency of co-occurrence indicated a closer relationship between the keywords. However, the absolute value represented by the frequency cannot reflect the true interdependence between them in an actual econometric analysis. Therefore, we adopted the Ochiai coefficient (also known as the normalized coefficient) to obtain the correlation matrix [27], and then produced a dissimilarity matrix-form correlation matrix through the minus “1”.

(2) $$H=\frac{C_{ij}}{\sqrt{C_{i}\times C_{j}}}$$

Note. H represents the correlation between two high-frequency keywords, $C_{ij}$ represents the co-occurrence frequency between i and *j*, $C_{i}$ represents the frequency of keyword i, and $C_{j}$ represents the frequency of keyword j.

**Figure 4:**
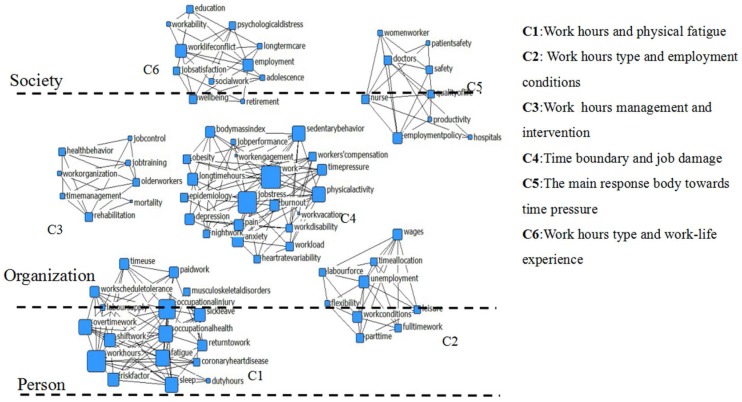
The hierarchical structure of cluster analysis.

Systematic cluster analysis (software: SPSS 19.0; method: ward; metric: squared Euclidean distance) was used to analyze the dissimilarity matrix. Figure 4 presents the six clusters which were named according to their special study content: work hours and physical fatigue (C1); work-hours type and employment conditions (C2); work-hours management and intervention (C3); time boundary and job damage (C4); the main response body towards time pressure (C5); and work-hours type and work-life experience (C6). We located each cluster in the hierarchy of individual–organization–society integration, and demonstrated that there were cross-level clustering variables in C1, C2, C5 and C6. Importantly, clusters are not absolutely independent from each other; along these lines, our goal was to provide clear and unbiased information for the current distribution properties of work hours.

## 4. Discussion

### 4.1. Map Changes under the Timeline

Although the study theme of work hours demonstrated frequent change from the organizational perspective throughout the targeted period, there were still some classic topics that prevailed at the individual level, and alternated consistently among fatigue, leisure and sleep, while psychological distress began to emerge in 2010. Research from a social perspective was lacking in systematization and coherence in time cues, and has recently focused on work–life conflicts.

#### 4.1.1. The Statement of the Research Status during 1901–1989

During 1901–1950, the happened World Wars disrupted the world economy which took Europe as the economic core, and the fluctuating world economy, in turn, influenced work hours which were sensitive to such fluctuations. As a result, the direct consequence was unstable wage changes attached to the work hours. Especially after the World War II, the economic recovery of Europe and Japan pushed the female workers into the labor market which shocked the traditional paradigm of gender roles, and which also set off an upsurge on studying work preference of female workers [28]. What is more, after this, gender difference became a research topic [29]. Then, the economic competition pressure promoted the upgrade of the organization, and also spawned more flexible and diversified work types such as part-time work and flexibility when considering personalized needs, as well as the staff enthusiasm in management. In this case, employees could be encouraged to work voluntarily.

After 1950, the ideal of requesting personal self-development became significantly influenced by the “social man” and the “self-realization” hypothesis of human nature [30,31], and people also suffered a sense of being bound by work hours. Indeed, individual-oriented research gradually attracted attention when the early studies on fatigue and leisure emerged 

At the same time, the doctors, as the most sensitive group towards work hours, were also brought to the public view. This was partly because of the rapid population increased the workload and the emotional labor of doctors after World War II.

Evidence shows that these factors aggravate burnout in doctors [32,33]. In addition, many hospitals were exploring new practice for improving service quality, such as implementing “no holiday in hospital”, which required hospitals to be open throughout the day, even on weekends and holidays [34]. These schedules irregularly extended doctors’ work hours and interrupted the life rhythm, leading to the potential worsening of doctors’ physical and mental health [35].

#### 4.1.2. The Statement of the Research Status during 1990–1999

With the profound changes in the political and economic map of the world around 1990, which accelerated competition and cooperation among countries, the organization, as the basic element of economic development and the most important mainstay of the market, exerted more pressure for overtime work [36]. This dynamic caused an upgrading contradiction between individuals and organizations, leading to the emergence of shift work which was an improvement in job life and leisure time [37]. Meanwhile, the concentration of work hours has turned from traditional economics to sociology—from traditional work elements (wages, vacations, etc.) to individuals’ work experience, such as job stress and overtime, which are still being explored [38]. Furthermore, this situation spawned research on protective factors such as physical activity to mitigate the conflict between individuals and organizations [39]. At the same time, some keywords like “sick leave” and “occupational health” surfaced, which reflect the negative impact of work hours on individual employees [40].

#### 4.1.3. The Statement of the Research Status during 2000–2010

With the in-depth study on the individual–organization, occupational injuries, which represent the negative impact of work factors on individuals, became the core issue of work-hours research, along with job training and employment. Occupational injuries were classified into two categories: acute and chronic [41], and the damage caused in work hours for employees mainly extended exposure time to hazards, thereby enhancing the possibility and severity of work hazards [8]; in addition, along with establishment of labor law, the negotiation between organizations and unions, and even governments, promoted the stability of employment influenced by the social contract theory, and eased the work-hours tension [2,42].

Meanwhile, the nurses, due to the combination of the gender characteristics of female workers and the work characteristics of doctors, also attracted social attention, partly caused by the high work pressure, the complicated work environment, and the low social status of nurses. The nurses were trained to assist doctors with the instrumental and emotional support such as “caring for the sick” or “executing the doctor’s advice” in the traditional social structure [43], and their social benefits were far fewer than those of doctors. Indeed, the female workers still have not been widely recognized, and thus the occupational injury of nurses became heavier [44].

#### 4.1.4. The Statement of the Research Status after the 2010s

After 2010, the research mainly focused on long work hours and the psychological distress, as well as work–life conflict. As an important stressor for physical and psychological problems, long work hours could induce the individual into an unhealthy lifestyle (e.g., smoking, alcohol abuse and harmful dietary regimens) [45], and could contribute to the development of cancer [46]. Obviously, the economic benefits of long work hours are much lower than the social costs they need to consume. Another emerging research theme was the psychological distress from the individual perspective, which was caused by the management practice of the frequent burnout, depression and suicide [47]. Indeed, the research on work–life conflict from the social perspective has become a salient topic.

### 4.2. Exposition of the Research Situation from the Perspective of the Study Theme

#### 4.2.1. Work Hours and Physical Fatigue

The relationship between the individuals’ work hours and their physical fatigue was the core theme of this cluster. The results demonstrated that some keywords with time characteristics of the length or span, such as overtime work, shift work and work-schedule tolerance, were significantly associated with the keywords such as coronary heart disease, sick leave, sleep and fatigue—which are associated with physical fatigue. This suggests that long or irregular work could aggravate serious physical fatigue, significantly disrupt sleep quality, or result in sick leave or absenteeism [48,49]. Specifically, the co-occurrence of shift work and work-schedule tolerance indicated individuals were suffering from the negative effects of high-frequency conversion. Overtime work refers to employees’ actual work hours beyond their employers’ stipulation [50]. According to the self-loss theory, overtime could accelerate fatigue [51], compromise sleep quality [52], be closely associated with hypertension or cardiovascular disease [53], and increase consumption of limited resources.

#### 4.2.2. Work-Hours Type and Employment Conditions

Here, the relationship between employment conditions of organizations and work hours is mainly discussed. The organization-related characteristics, such as work conditions, part-time or full-time status, and flexibility, were closely related to employees’ time allocation, leisure activities and wages. Specially, there were more comparative studies on part-time and full-time work, suggesting the rise in popularity of this area [54]. The reason for this phenomenon was partly that the majority of women workers value flexible, part-time jobs, given the demand for combining a career with being a mother or spouse, and high-speed operation of the world’s economic development system which stimulates the organization’s demand for more flexible labor, or short-term labor, which could also create more opportunities for part-time workers [55].

#### 4.2.3. Work-Hours Management and Intervention

After realizing the passive consequences resulting from long work hours, some researchers and organizations have begun to explore protective factors to solve or alleviate this conundrum. For instance, time management was proposed to promote individual efficiency in time use by making rational plans and controlling overtime work [56]. Moreover, the scope of time management has extended to so-called “life management”, with more general consideration than the traditional “eight-hours management” in the industrial revolution [57]. Also, the intervention could contribute to maintaining a work–life balance, as well as boosting productivity, such as in job training, seminars, salons and other exchange programs [58]. Meanwhile, there were also some interventions providing life skills to improve staff’s family life wisdom or skills [59]. What is more, some data showed that enhancing job control, promoting positive work experiences, and encouraging employees to develop healthy behaviors, could relieve employees’ psychological stress and improve employees’ overall health [60].

#### 4.2.4. Time Boundary and Job Damage

According to the boundary theory, individuals drew a line between home and work, and thus the individual activities were separated into independent and interrelated realms [61]. However, the significant relationship between work vacation and long work hours reflected a phenomenon that the irregular shift or even the fuzzy work–life boundary has appeared because of rapid life rhythms and varied position types. This irregular shift not only caused physiological disruption, but it also contributed to decreasing happiness [62], suffering burnout [63], exacerbating depression, and even causing suicide [64].

#### 4.2.5. The Main Response Subjects toward Time Pressure

Present research demonstrates that the main individuals experiencing time pressure were doctors, nurses and women workers. It is well known that doctors were the main victim as they suffered severe time pressure [65], as do nurses [66]. Aside from overtime work, long work hours and irregular routines, doctors and nurses were also required to express emotional labor as part of doing “people work” [67], and those people are more likely to exhaust themselves. The women workers also experienced significant time pressure [68]. It has been previously reported that females with long work hours have a higher divorce rate than those with shorter hours [69]. Affected by the traditional culture, which requires women to care for their families, female workers were forced to meet familial demands and organizational requirements. Hence, women experienced more conflict due to the unbalanced work hours, which would result in more physiological and psychological problems [70].

#### 4.2.6. Work-Hours Types and Work-Life Experience

Cousins and Tang pointed out that males and females both faced serious work–life conflict because of long work hours [71,72]. The extended work-hours boundary disrupted the original work–life balance, and people wandered between work and life, forced to shuttle between and mould two realms, which could in turn lead to more serious psychological conflict, or even disability retirement [73]. According to the role theory, an interactive role conflict will occur when confronting distribution contradictions between work and life in time, space, emotion, energy or materials [74]. Some researchers have also found that the psychological tension caused by time pressure is a decisive factor in family conflict [75,76]. Fortunately, the stress may be alleviated by strengthening the individual’s sense of work control [58,77]. Other studies have explored factors in buffering work–life conflict, such as flexible work schedules [78], an inclusive family atmosphere [79], high-quality social support [80,81], and high occupational interactional requirements [82].

## 5. Conclusions and Future Directions

### 5.1. Conclusions

Multilevel and cross-time analysis methods could make research more comprehensive and accurate. Our paper untangled 6364 pieces of work, produced between 1901 and 2017, from the Web of Science, related to work hours, and extracted 77 high-frequency keywords. Conclusions are drawn below:

(1) The analysis of publications showed that the amount of research related to work hours is fluctuating upward, and there has been considerable growth in recent years. Relevant journals with work-hours research were mostly concentrated in the fields of medicine, sociology and management science. In the range of journals with over 50 relevant articles, the BMJ topped the list with more than 200 published pieces.

(2) The social network diagram of high-frequency keywords showed that work, work conditions, job stress, and work–life conflict were the core issues on the map of work hours. According to the time sequence, further analysis based on the individual–organization–society integrative perspective demonstrated that the time inflection of work hours on the organizational level appeared in approximately 1990, and the themes here ranged from the early economic perspective to the social perspective with the attention on organizational factors to work experience to explore the impact on employees caused by work hours and other organizational factors. Of note, the theme of long work hours has recently entered the research mainstream; there were no significant changes on the individual level, with obesity, fatigue and sleep quality being the main study themes. In addition, burnout and psychological distress have gradually become the focus of research from the individual perspective; prior studies focused on the individual or organizational levels rather than systematically on the social level. The research achievements of the social perspective lack the focus on present themes, such as work–life conflict.

(3) This paper provided a better interpretation for the panorama of work hours by tracing and classifying the study themes according to time. The cluster analysis of high-frequency keywords showed these six clusters: (C1) work hours and physical fatigue; (C2) work-hours types and employment conditions; (C3) work-hours management and intervention time boundary; (C4) job damage; (C5) the main response body towards time pressure; and (C6) work-hours types and work-life experience. Further, more network nodes contained in C1 and C2 indicated that the current research on those types are quite abundant, while fewer nodes contained in C3 and C5 indicated deeper exploration in those fields. In addition, the cluster analysis based on the individual–organization–society integration perspective illustrated a multilevel, amalgamated trend as the cross-level cluster variables contained in C1, C2, C3, C5 and C6.

### 5.2. Future Directions

#### 5.2.1. The Production Mechanism and Intervention Measures of Organizing Long Work Hours

Long work hours have been the normality for employees and the common way of organizations [83]. However, previous studies only focused on the direct consequence caused by long work hours, and treated it as an independent variable to examine its impact on individuals, organizations or society [84,85]. Few researchers have attempted to discuss the formative mechanism behind this dynamic. Importantly, the negative effects of long work hours would diffuse within the organization and increase the likelihood of employees experiencing economic and psychological burden. Thus, the mechanism of long work hours needs to be examined based on organizational and individual clues. Accordingly, the research and excavation of a reasonable intervention mechanism to reduce time burden has significant theoretical and practical value. Further, it is also a potent research field which may make contribution to balance the environmental change, organizational competition pressure, and individual health, as well as life needs (through the innovative work mode and system design based on the negative effects being addressed).

#### 5.2.2. The Formation Mechanism of Individual Work-Hours Motivation and Its Relationship with the Perception of Physical and Mental Health

It is essential to analyze the individual motivation towards work hours. The work of exploring work-hours motivation has great significance in understanding the psychological mechanisms as well as the behavioral differences associated with long work hours. In addition, different work-hours motivations characterize the acceptable length of work boundary, which is closely related to physical and mental health status; however, we must also ask, why are some individuals satisfied or content with their long work hours, while others may feel great unease [86]? There are significant differences between individuals’ perception of physical and mental health, driven by different motivations. The relationship between work hours and physical and mental health, in consideration of individual motivation, needs to be further investigated. Future research in this space could also introduce environmental variables [86], such as monitoring individual responses on long work hours under different cultures and motivational dispositions, so as to further understand and forecast their behavior and associated outcomes.

#### 5.2.3. The Dynamic Changes of the Individual Time View under the New Social Background

In the context of economic globalization, increased social wealth has not only provided mankind with abundant material resources, but also reduced people’s dependence on human economic activities. Further, people’s values and life concepts have undergone substantial changes, including greater attention to physical health and happiness, and the emergence of the so-called “end of the work” [87]. Thus, the oft-contradictory relationship between money and health is gradually manifesting. Do members of society generally prefer to acquire wealth by sacrificing health through long work hours, or do they support a rational work schedule to maintain a healthy work–life balance? This complex issue will encourage people to rethink their lifestyles and life concepts. In sum, for the public, the meaning of long work hours should be re-examined in the context of social change, and exploring other contours of time is equally paramount.

## Figures and Tables

**Table 1:** Top 10 journals that have published at least ten research articles.

Journal	Number	Journal	Number
British Medical Journal	210	Gerontologist	63
International Labour Review	156	Time & Society	58
Journal of Occupational and Environmental Medicine	146	Work Employment and Society	55
Monthly Labor Review	97	Ergonomics	53
Scandinavian Journal of Work Environment & Health	94	Chronobiology International	48

**Table 2:** Top 20 high-frequency keywords related to work hours.

Rank	Keywords	Frequency	Rank	Keywords	Frequency
1	work hours	710	11	occupational injury	164
2	occupational health	430	12	flexible work hours	144
3	work	284	13	work life conflict	121
4	sleep	249	14	nurses	119
5	job stress	235	15	part-time work	109
6	employment	185	16	fatigue	101
7	sick leave	183	17	women	90
8	job training	182	18	long work hours	83
9	work disability	172	19	return to work	76
10	shift work	166	20	overtime work	73
